# Supplementary material for: Slow oscillation-spindle coupling predicts enhanced memory formation from childhood to adolescence
Source: eLife. 2020 Jun 24;9:e53730. doi: 10.7554/eLife.53730 (PMC7314542; doi:10.7554/eLife.53730)
Supplement: Transparent reporting form [file elife-53730-transrepform.pdf]

eLIFE
1st Floor
24 Hills Road
Cambridge CB2 1JP, UK
P 01223 855340
W elifesciences.org
T @elife

eLife's transparent reporting form

We encourage authors to provide detailed information *within their submission* to facilitate the interpretation and replication of experiments. Authors can upload supporting documentation to indicate the use of appropriate reporting guidelines for health-related research (see EQUATOR Network), life science research (see the BioSharing Information Resource), or the ARRIVE guidelines for reporting work involving animal research. Where applicable, authors should refer to any relevant reporting standards documents in this form.

If you have any questions, please consult our Journal Policies and/or contact us: editorial@elifesciences.org.

Sample-size estimation

- You should state whether an appropriate sample size was computed when the study was being designed
- You should state the statistical method of sample size computation and any required assumptions
- If no explicit power analysis was used, you should describe how you decided what sample (replicate) size (number) to use

Please outline where this information can be found within the submission (e.g., sections or figure legends), or explain why this information doesn't apply to your submission:

In this study we conducted a longitudinal experiment. Initial sample consisted of 63 subjects (cf. Hoedlmoser et al. 2014). We did not compute a sample size because the sample size was determined by the positive responding rate of the subjects who were willing to participate again in the current follow-up study.

Detailed description of the sample and how they were recruited can be found in the methods section in the Participants paragraph (p. 25).

Replicates

- You should report how often each experiment was performed
- You should include a definition of biological versus technical replication
- The data obtained should be provided and sufficient information should be provided to indicate the number of independent biological and/or technical replicates
- If you encountered any outliers, you should describe how these were handled
- Criteria for exclusion/inclusion of data should be clearly stated
- High-throughput sequence data should be uploaded before submission, with a private link for reviewers provided (these are available from both GEO and ArrayExpress)

Please outline where this information can be found within the submission (e.g., sections or figure legends), or explain why this information doesn't apply to your submission:

We did not perform any biological replicates.

For technical replicates we used bootstrapping. Details about the iterations and methodological considerations can be found in the corresponding paragraphs in the methods section (Event-locked time-frequency representations [p. 31], Event-locked Cross-frequency coupling [p.31] & statistical analyses [p.33])

Outliers were handled by computing non-parametric statistical analyses

Rationale for the exclusion of participants is stated in the Participants paragraph in the methods section (p. 25).

eLife Sciences Publications, Ltd is a limited liability non-profit non-stock corporation incorporated in the State of Delaware, USA, with company number 5030732, and is registered in the UK with company number FC030576 and branch number BR015634 at the address 1st Floor, 24 Hills Road, Cambridge CB2 1JP | August 2014

Statistical reporting

- Statistical analysis methods should be described and justified
- Raw data should be presented in figures whenever informative to do so (typically when N per group is less than 10)
- For each experiment, you should identify the statistical tests used, exact values of N, definitions of center, methods of multiple test correction, and dispersion and precision measures (e.g., mean, median, SD, SEM, confidence intervals; and, for the major substantive results, a measure of effect size (e.g., Pearson's r, Cohen's d)
- Report exact p-values wherever possible alongside the summary statistics and 95% confidence intervals. These should be reported for all key questions and not only when the p-value is less than 0.05.

Please outline where this information can be found within the submission (e.g., sections or figure legends), or explain why this information doesn't apply to your submission:

Statistical analysis methods are described in detail in the methods in the statistical analyses paragraph (p. 33) and briefly described in the main text as well. For every statistical method used, we referenced the corresponding figure where the results are displayed.

(For large datasets, or papers with a very large number of statistical tests, you may upload a single table file with tests, Ns, etc., with reference to sections in the manuscript.)

Group allocation

- Indicate how samples were allocated into experimental groups (in the case of clinical studies, please specify allocation to treatment method); if randomization was used, please also state if restricted randomization was applied
- Indicate if masking was used during group allocation, data collection and/or data analysis

Please outline where this information can be found within the submission (e.g., sections or figure legends), or explain why this information doesn't apply to your submission:

The longitudinal study design allowed us to exclusively use within-subject analyses. Therefore, we did not conduct any group allocation or randomization.

Additional data files ("source data")

- We encourage you to upload relevant additional data files, such as numerical data that are represented as a graph in a figure, or as a summary table
- Where provided, these should be in the most useful format, and they can be uploaded as "Source data" files linked to a main figure or table
- Include model definition files including the full list of parameters used
- Include code used for data analysis (e.g., R, MatLab)
- Avoid stating that data files are "available upon request"

Please indicate the figures or tables for which source data files have been provided:

Data Availability: Data required to reproduce the main conclusions and all figures are available at https://datadryad.org/stash/share/cfIM7ulW8stBlf9saY9fD1Zt-akK9oud-FVmXf_AbJo<br><br>doi:10.5061/dryad.8sf7m0chn (preliminary)
